# Mast Cells at the Crossroads of Hypersensitivity Reactions and Neurogenic Inflammation

**DOI:** 10.3390/ijms26030927

**Published:** 2025-01-23

**Authors:** Ernesto Aitella, Ciro Romano, Lia Ginaldi, Domenico Cozzolino

**Affiliations:** 1 Department of Clinical Medicine, Public Health, Life and Environmental Sciences, University of L’Aquila, 67100 L’Aquila, Italy; ernestoaitella@gmail.com (E.A.); lia.ginaldi@univaq.it (L.G.); 2 Allergy and Clinical Immunology Unit, “G. Mazzini” Hospital, ASL Teramo, 64100 Teramo, Italy; 3 Clinical Immunology Outpatient Clinic, Division of Internal Medicine, Department of Advanced Medical and Surgical Sciences, “Luigi Vanvitelli” University of Campania, 80131 Naples, Italy; ciro.romano@unicampania.it; 4 Division of Internal Medicine, Department of Precision Medicine, “Luigi Vanvitelli” University of Campania, 80131 Naples, Italy

**Keywords:** mast cells, allergy, IgE, neurogenic inflammation, MRGPRX2, cannabinoid receptors, MRGPRX2 antagonists

## Abstract

Although mast cells have long been known, they are not yet fully understood. They are traditionally recognized for their role in allergic reactions through the IgE/FcεRI axis, but different groups of surface receptors have since been characterized, which appear to be involved in the manifestation of peculiar clinical features. In particular, MRGPRX2 has emerged as a crucial receptor involved in degranulating human skin mast cells. Because of mast cells’ close proximity to peripheral nerve endings, it may play a key role in neuroimmune interactions. This paper provides an overview of mast cell contributions to hypersensitivity and so-called “pseudoallergic” reactions, as well as an update on neuroinflammatory implications in the main models of airway and skin allergic diseases. In particular, the main cellular characteristics and the most relevant surface receptors involved in MC pathophysiology have been reappraised in light of recent advancements in MC research. Molecular and clinical aspects related to MC degranulation induced by IgE or MRGPRX2 have been analyzed and compared, along with their possible repercussions and limitations on future therapeutic perspectives.

## 1. Introduction

Mast cells (MCs) are strategically located at the body’s boundaries, where they can react upon exposure to infectious agents, alarmins, and danger signals or take part in both innate and acquired immunity or immune tolerance responses in the context of respiratory, cardiovascular, gastrointestinal, and skin systems, as well as in the settings of cancer, autoimmunity, allergy, and mastocytosis [1,2]. The main expression of this versatility is represented by the multitude of surface receptors that mirror their functional potential and ability to respond to various stimuli. Another characteristic feature is the phenotypic diversity of MCs, which typically reach maturation in tissues, as seen in the case of mucosal MCs (MMCs) and connective tissue MCs (CTMCs) in rodents. This heterogeneity also corresponds to plasticity linked to MCs’ ability to respond and diversify according to the cytokine environment, sensor molecules, and cell surface receptors and to the possibility of “transdifferentiating” from one phenotype to another [3].

**Figure 1:**
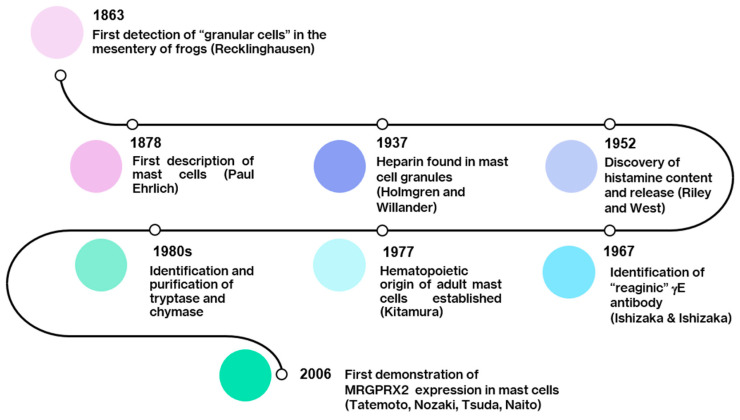
Timeline of main historical events of mast cell research.

Historically, MCs were first characterized morphologically because of their fascinating content in granules and typical metachromasia. Subsequently, functional studies highlighted their ability to degranulate in response to so-called allergic stimuli (Figure 1). Due to several problems in setting up reproducible and reliable studies, MC research proceeded slowly until the advent of cell lines and alternative MC-deficient mouse strains. Only relatively recently, particularly with the reappraisal of the Mas-related G protein-coupled receptor member X2 (MRGPRX2), has the indissoluble “MC/IgE” binomial of allergic reactions been questioned, unveiling an interesting dichotomy comprising both IgE and non-IgE mediated mechanisms underlying MC reactivity and providing an explanation for some pathophysiological mechanisms the FcRI/IgE paradigm alone could not account for [4]. Additionally, MCs located close to nerve endings have been found to interact with the nervous system in a broad neuroimmune cross-talk through their own mediators or in response to neuromediators, such as substance P (SP) or other tachykinins [5,6].

This review focuses on updating the understanding of mast cell functional complexity, merging and blending allergy and pseudoallergy mechanisms, with attention to the intriguing scenario of neurogenic inflammation and its implications. In particular, the main cellular characteristics and the most relevant surface receptors involved in MC pathophysiology have been reappraised in light of the recent advancements in MC research. The molecular and clinical aspects related to MC degranulation induced by IgE or MRGPRX2 have been analyzed and compared, with their possible repercussions and limitations on future therapeutic perspectives.

## 2. Mast Cells: Overview

MCs are bone marrow-derived and tissue-resident immune cells, particularly dependent upon the stem cell factor (SCF) for their survival [7]. They were first described by Paul Ehrlich in 1878 as cytoplasmic metachromatic granulated cells. Metachromasia occurs when a structure assumes a different color compared to the dye itself due to the presence of polyanions and the polymerization of dye molecules, resulting in a shift in the absorption spectrum of the dye and a hypochromic color change. In the case of MCs, the main stains are represented by Wright, Giemsa, May-Grünwald Giemsa, and Leishman included in the Romanowsky staining or toluidine blue, causing them to acquire a red-to-purple coloration of their granules [8].

In detail, MCs are enriched in heparin and serglycin, a proteoglycan. Histamine is the main biogenic amine described in MCs, whereas tryptase and chymase are two of the best-known proteases. Among other preformed mediators, there are lysosomal enzymes such as β-hexosaminidase, β-glucuronidase, and cathepsins; the proteases carboxypeptidase A, granzyme B and matrix metalloproteinases; cytokines, chemokines, and growth factors; other proteins, enzymes, and peptides such as heparanase, angiogenin, active caspase-3, corticotropin-releasing hormone, endorphin, endothelin-1, SP, vasoactive intestinal peptide, and eosinophil major basic protein [9].

The preformed granules and the neoformed and neosynthesized mediators are important to understanding the role of MCs in early, late, and chronic phases of their actions and functions. In fact, among arachidonic acid derivatives, neoformed eicosanoids from phospholipase A2, converted into prostaglandin D2 by cyclooxygenase and into leukotrienes by 5-lipoxygenase, contribute to the recruitment of immune effector cells, vascular permeability, smooth muscle contraction, mucus production, and activation of sensory neurons [10,11,12]. Nerve growth factor, platelet-derived growth factor, vascular endothelial growth factor (VEGF), and fibroblast growth factor-2 are known to play roles in homeostasis, wound healing, and tissue repair; histamine, tryptase—via protease-activated receptor-2 (PAR-2)—and osteopontin have been implicated in bone remodeling [13,14,15,16].

**Figure 2:**
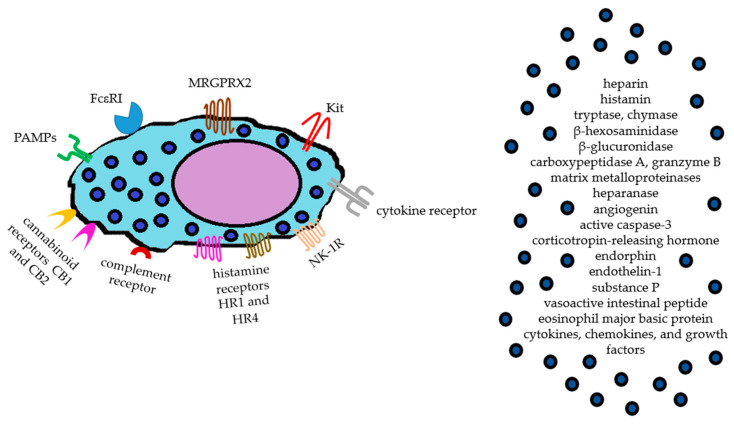
Main mast cell surface receptors and list of relevant preformed mediators found in mast cell granules.

Histamine, tumor necrosis factor α (TNF-α), interleukin (IL)-10, pro-angiogenetic factors, and proteases favor tumor progression and metastasis, whereas tryptase and IL-5 appear to exert anti-neoplastic effects [17]. On the other hand, included among the groups of surface receptors of human MCs are high-/low-affinity FcεRI and FcεRII receptors of immediate allergic reactions, respectively; FcγRIIa; kit receptor or CD117 with affinity to SCF; G protein-coupled receptors (GPCRs) as well as histamine receptors type 1 and 4 (H1R, H4R), complement component C3a receptor, cannabinoid receptor type 1 and 2 (CB1 and CB2), and MRGPRX2 [18] (Figure 2).

In particular, Toll-like receptors (TLRs), NOD-like receptors (NLRs), and retinoic acid-inducible gene 1 (RIG-1)-like receptors (RLRs) are pathogen-associated molecular patterns (PAMPs) receptors that are linked to innate immunity in response to Gram-positive and Gram-negative bacteria and Mycobacterium tuberculosis. MCs are also involved in parasitic infections and, although less characterized, in viral infections such as herpes simplex virus 2 or as long-lived HIV viral reservoirs [19,20]. They are also capable of phagocytosing bacteria, producing reactive oxygen species, and releasing MC extracellular traps. Regarding adaptive immunity, they have been demonstrated to be endowed with the ability to process and present antigens via MHC class I and class II complexes, to modulate the activation of dendritic cells (DCs) and their migration to lymph nodes to induce Th2 responses, and to activate T lymphocytes by TNF-α. Conversely, MCs may also have immunosuppressive effects and, in particular, can induce the development of regulatory CD4+ CD25+ Foxp3+ T cells [9].

MCs have been associated with several dysreactive or autoimmune conditions or involved in experimental models of allergic encephalitis due to their location inside the blood–brain barrier (BBB) and the possibility of altering BBB permeability, interacting with brain cells, and inducing neuroinflammation [21]. Enhanced MC activity is also documented in the gut in inflammatory bowel diseases [1,22].

A crucial landmark in MC features is their widespread distribution at the interface of the host and the environment, particularly around blood vessels and nerves. They are found in connective tissue or sub-epithelial regions of the respiratory mucosa, gastrointestinal tract, heart, and other organs, while in the skin, they are resident, long-lived cells. Furthermore, N-cadherin or synaptic cell adhesion molecules define synaptic-like structures between MCs and neurons. Unlike MMCs and CTMCs in rodents, among human mature MCs, MCs containing tryptase, chymase, carboxypeptidase, and cathepsin are prevalently found in the skin, whereas MCs expressing only tryptase are represented in the lung and gut.

Regarding the skin, MCs are usually located within the dermis and near the nerve endings in the epidermis and have also been detected in the epidermis in psoriasis [23]. Such localization represents the rationale for the methodology of skin prick testing as a diagnostic tool in hypersensitivity to common airborne allergens, food allergens, hymenoptera venoms, and drugs [24].

## 3. IgE/FcεRI Axis

**Figure 3:**
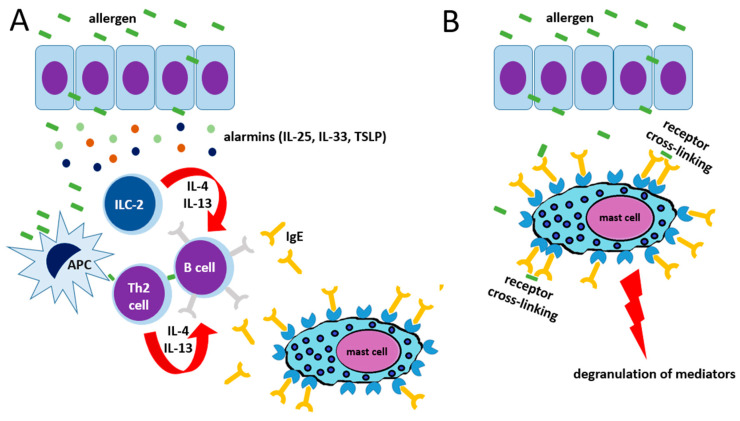
(**A**) Sensitization phase. Allergens penetrate through the epithelial surface, triggering the release of alarmins, which start the type 2 inflammation program by inducing type 2 innate lymphoid cells (ILC-2) to release interleukin(IL)-4 and IL-13 and favoring T helper 2 (Th2) cell development by antigen-presenting cells (APCs). IL-4 and IL-13 drive an isotype switch towards IgE in B cells, with consequent release of IgE able to bind high-affinity receptors (FcεRI) on mast cells. (**B**) Effector phase. Subsequent exposure to the same allergens directly triggers receptor cross-linking via IgE binding on the mast cell surface, leading to cell degranulation and the consequent clinical effects depending on the target organ involved in the hypersensitivity reaction.

Type 1 hypersensitivity, according to Gell and Coombs, is the consequence of the binding of IgE antibodies to the high-affinity receptor FcεRI on previously sensitized MCs and basophils. During the sensitization phase, in genetically predisposed individuals and the presence of favorable environmental factors, the altered leaky epithelial barrier and its exposure to parasites, bacterial toxins, viruses, protease activity of allergens, and air pollution amplify the damage to the epithelium and the production of chemokines, cytokines, and alarmins such as IL-25, IL-33, and thymic stromal lymphopoietin (TSLP). This activates innate lymphoid cells 2 and DCs. After antigen presentation by DCs, naïve T cells (Th0), in such a Th2 cytokine environment, differentiate into IL-4/IL-13 producing Th2 cells that, through CD40/CD40L interaction, induce the switch of B cells into IgE-secreting plasma cells. Then, IgE binds FcεRI on MCs and basophils, which degranulate after subsequent exposure to multivalent allergenic antigens and crosslinking [25] (Figure 3).

At this point, a rapid release of pre-formed mediators occurs within minutes and newly synthesized ones within 15 min, resulting in the well-known effects of vasodilation, bronchoconstriction, and allergic inflammation, which characterize allergic rhinitis and asthma, food allergies, allergic reactions to venoms or medications, and more generally, IgE-mediated forms of urticaria-angioedema and anaphylaxis.

From a mechanistic point of view, introducing the time variable in these pathophysiological processes of granule release plays a decisive role in understanding and managing events such as fearsome biphasic anaphylaxis [26]. Moreover, among its mediators, TNF-α acts as a chemoattractant for other immune effector cells, including neutrophils and eosinophils, leading to an amplification of allergic inflammation and, if allergenic exposure and epithelial damage persist, to the development of a chronic inflammatory state with tissue remodeling or fibrosis [27].

During anaphylactic reactions, serum tryptase typically exceeds 120% of baseline (1.2 × baseline level μg/L + 2) and represents a reliable marker of MC degranulation, with a sensitivity and specificity for diagnosis of 73% and 91%, respectively, if blood withdrawal is carried out preferably 30 min after the event and within 6 h. Even if an age progression has been shown, basal serum levels between 8 and 11 μg/L should be considered with caution and potentially linked to hereditary alpha-tryptasemia (HαT), while levels above 20 μg/L may be suspicious for mastocytosis [28]. Histamine, as a preformed and pivotal mediator, is not used in clinical practice to measure MC degranulation due to its short half-life; however, flow cytometry detection of CD63 externalization induced by a culprit allergen may be performed as an MC activation test assay and as the basophil activation test for basophils [29].

## 4. Pseudoallergy

**Figure 4:**
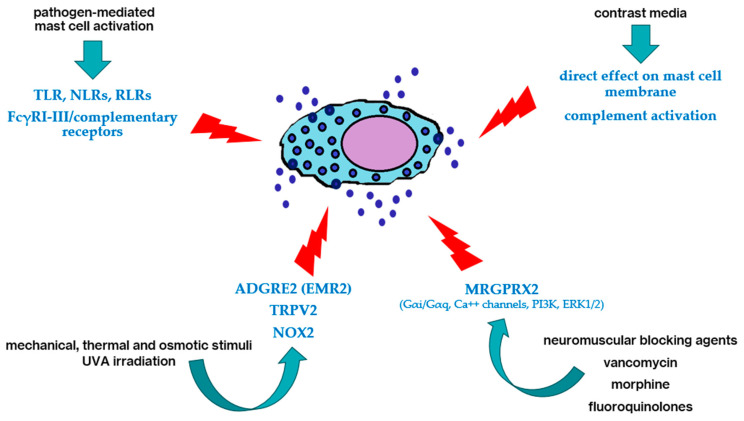
Examples of non-IgE-mediated (i.e., “pseudoallergic”) pathways of mast cell degranulation (details in the text).

Pseudoallergy is a nonallergic form of hypersensitivity, clinically similar to immediate-type allergic reactions without evidence of IgE-mediated mechanisms [30]. Independently of IgE/FcεRI interaction [31], MCs have also been shown to degranulate due to different stimuli (Figure 4). Pathogen-mediated MC activation through TLR, NLRs, RLRs, and FcγRI-III/complementary receptors may lead to MC degranulation, involving alternative mediators to histamines such as platelet-activating factor (PAF). This may explain why infections may be complicated by acute urticaria in an IgE-independent manner [32]. Indeed, studies on the pediatric population with a history of skin involvement during infectious events and concomitant treatment with beta-lactams showed that only a small proportion of children tested positive in drug provocation tests [33].

MC releasability is particularly implicated in adverse reactions to contrast media, where degranulation may be due to a direct effect on the MC membrane or direct complement activation [34,35]. Notably, in a recent prospective study about hypersensitivity to iodinated contrast media, skin tests against the causative agent were negative in 66.3% of patients, suggesting the involvement of alternative pathways [36].

Furthermore, in response to physical stimuli, for example, in inducible urticaria, non-IgE-mediated mechanisms are known to be triggered, involving adhesion G protein-coupled receptor E2 (ADGRE2 or EMR2) and ion channel TRPV2 for mechanical, thermal, and osmotic stimuli; NOX2 in response to UVA irradiation; suppression of tumorigenicity 2 (ST2) and P2X1 receptors for alarmins. Interestingly, the ADGRE2 C492Y mutation is linked to autosomal dominant vibratory urticaria and HαT [37,38].

Another area that does not completely fit the IgE model is represented by the adverse reactions to neuromuscular blocking agents, which complicates the allergy workup of perioperative adverse reactions [39]. Some studies have already shown discrepancies between self-reported allergy to fluoroquinolones and negative skin tests or non-reproducible drug provocation tests, even in the presence of comorbidities or after correction for concomitant gastroesophageal chemical-infective inflammation [40,41,42].

These considerations lead to the inclusion of alternative pseudoallergic degranulation pathways of MCs, such as via MRGPRX2 (Figure 4). In particular, cation groups of vancomycin, atracurium, mivacurium, cisatracurium, rocuronium, morphine, and fluoroquinolones may interact with the drug binding site for MRGPRX2, including a tetrahydroisoquinoline motif. Wang et al., in MCs purified from human skin, recently studied MRGPRX2 activation by agonist compound 40/80 and SP, in the presence or absence of inhibitors, and FcεRI aggregation, highlighting the requirements of Gαi more than Gαq, the crucial role of Ca++ channels and PI3K, and the additional participation of ERK1/2 in MRGPRX2-mediated degranulation. Interestingly, in FcεRI aggregation, despite the activation of similar kinases but with distinct dependence on Ca++ for AKT phosphorylation, the time-course was 8–30 min compared to the rapid response of MRGPRX2-elicited degranulation within around 1 min [43].

However, MRGPRX2 seems to correlate more with mild-to-moderate events in drug-naïve patients, with resolution after a decrease in drug concentration below EC50, particularly in the presence of eosinophil/basophil-driven comorbidities or in chronic urticaria, where MRGPRX2-positive cells are increased [44]. Like acute conditions, chronic urticaria is being approached outside the IgE-FcεRI axis, exploring comorbidities in the context of neuroimmune pathways as well as the brain–gut-skin axis in gastrointestinal complaints and the Th2-like profile overlap syndrome of urticaria and gastroesophageal reflux [45,46,47].

## 5. Organ-Specific Features

In humans, at least six different transcriptomic clusters of MCs across 12 organs have been identified [48,49]. In particular, MCs are physiologically distributed in the human heart in the myocardium and pericardium, aortic valve, and adventitia of arteries. They have been shown to be involved in cardiovascular disorders such as atherosclerosis, myocardial infarction, myocarditis, hypertension, heart failure, and cardiac remodeling and fibrosis through fibroblast growth factor, TGF-β, or ACE-independent chymase pathways [50]. As a “shock organ”, the heart may be involved in anaphylaxis with hypotension, angina, cardiac arrhythmias, myocardial infarction, or cardiac arrest [51]. A rarely diagnosed, life-threatening form of hypersensitivity affecting the heart is known as “Kounis syndrome”. In this syndrome, allergic angina and allergic myocardial infarction are characterized by vasospasm-induced coronary blood flow reduction during allergic, anaphylactic, or anaphylactoid reactions as a consequence of MC degranulation. It manifests as coronary artery spasm, myocardial infarction, or stent thrombosis and may not respond to antiallergic therapies, requiring acute coronary syndrome treatment protocols [52].

Regarding the skin, despite the forms of urticaria triggered by IgE or non-IgE mediated mechanisms, MCs can be involved in cutaneous manifestations in the course of mastocytosis, with maculopapular cutaneous mastocytosis—previously known as urticaria pigmentosa—isolated or multifocal cutaneous mastocytomas, and diffuse cutaneous mastocytosis [53]. Mastocytosis is generally sustained by a KIT D816V mutation or other activating KIT mutations and/or in the context of HαT or clonal MC activation syndrome (MCAS) [54,55]. In mastocytosis, MCs may be responsible for allergic and/or hematologic manifestations, such as idiopathic anaphylaxis, bone marrow mastocytosis, smoldering systemic mastocytosis, systemic mastocytosis with associated myeloid neoplasm, MC leukemia, or the rare MC sarcoma. Interestingly, the allergy work-up for some reactions to hymenoptera venom or fluoroquinolones may show negative skin tests, suggesting the possible involvement of non-IgE mediated pathways, such as MRGPRX2-induced MC degranulation [56].

## 6. Mast Cells and Neurogenic Inflammation: From Lung to Brain

Neurogenic inflammation results from nerve activation, consequent neuropeptide release, rapid plasma extravasation, and edema [57]. In its original definition, it involves plasma leakage from the post-capillary venules of the skin and airways in response to stimulation of peripheral nerve endings. SP is considered the primary neuropeptide mediator released by non-myelinated sensory nerve fibers sensitive to capsaicin. It can induce focal, transient, and reversible gaps in the intercellular junctions of endothelial cells and interact with neurokinin 1 (NK-1) receptors. Among tachykinins, neurokinin A can induce vasodilation, plasma exudation, bronchoconstriction, and mucus secretion in the airways [58,59,60].

The background of neurogenic inflammation in the airways lies in the exposure of sensory nerves to the inflammatory environment following epithelial damage and the reduction in endopeptidases from epithelial cells and their ability to counteract the effects of tachykinins. In particular, SP has been shown to increase in the serum of asthmatic patients and to induce Th2 and MC-dependent lung inflammation in mice [61], while gabapentin has demonstrated the ability to alleviate cough hypersensitivity and neurogenic inflammation in a guinea pig model [62]. Conversely, the Th2 inflammatory environment seems to suppress ion channel TRPA1 expression in lung epithelial cells, which is increased by IFN-γ [63].

Moreover, MCs in the lung seem to be related not only to asthma but also to chronic obstructive pulmonary disease, respiratory infections, interstitial lung disease, and lung fibrosis. Their microlocalization within the lung may condition the multifunction and heterogeneity of MCs through cell–cell contact and adhesion signals, with immune-modulatory, proinflammatory, or pro-fibrotic activities both physiologically, in the defense against respiratory infections and pathologically, in chronic diseases. Furthermore, alveolar MCs, lacking the high-affinity IgE receptor, may transdifferentiate into a FcεRI-expressing phenotype in uncontrolled asthma [64,65]. In the context of the interaction of upper and lower airways in united airway disease, chronic rhinosinusitis, and nasal polyposis are risk and aggravating factors for asthma, where the finding of MCs in nasal cytology in non-allergic rhinitis infiltrated by eosinophils and MCs (NARESMA) is associated with both nasal polyps and asthma [66,67,68].

On the other hand, in a neuroimmunoendocrine organ such as the skin, the neuroanatomophysiological basis of neurogenic inflammation is constituted by the close connection between endothelial cells, keratinocytes, Langerhans cells, melanocytes, fibroblasts, and MCs with the peripheral nerve endings and their cross-talk via neurotrophins and neuropeptides [69]. As early as the late 19th century, experimental stimulation of dorsal roots showed vasodilation due to SP and calcitonin gene-related peptide (CGRP) released by nociceptors, with a supposed direct action on vascular endothelial cells and consequent increased permeability and neurogenic inflammation [70,71].

Thus, the synaptic-like connections between immune cells and nerve endings may link distant organs and foci of inflammation through nerves. Common examples of this communication are represented by viscero-somatic cross-sensitivity and the “convergence-projection” phenomena experienced in the referred thoraco-upper abdominal skin from heart and esophagus/stomach pain [72,73,74] or the neurogenic interconnection between the bladder and colon in interstitial cystitis [75]. In addition to peripheral phenomena, neurogenic inflammation can proceed at a central level with mechanisms of central sensitization up to phenotypic modifications of the nerve fibers. The main relevant consequence of this progression is the persistent modifications of neuroexcitability even when the stimulus is exhausted, with persistence and maintenance of the inflammation [76,77].

Clinical manifestations of this condition are represented by the phenomena of allodynia, alloknesis in chronic dermatitis and atopic dermatitis (AD), allosternutation in chronic rhinosinusitis with a primary allergic component, allotussivity in chronic cough and allergic bronchial asthma, cross-organ sensitization [78,79,80]. In the central nervous system, neurogenic inflammation may induce brain inflammation due to the presence of MCs close to the meninges, neurons, and microglia, clinically manifesting with headaches, “brain fog”, or neuropsychiatric disorders [81].

## 7. Mast Cell Skin Involvement and Neurogenic Inflammation

Neurogenic inflammation in the skin starts as a physiological process to maintain skin homeostasis against physical, chemical, and biological signals. Considering the contribution of serotonin to the behavioral functions of the hippocampus [82], it can even influence avoidance behavior, as seen in mast-cell-mediated antigen-avoidance behavior in response to the ingestion of allergens or toxins [83]. Instead, the increased expression of neurotrophins and peptidergic nerve fibers supports the neuroinflammatory contribution in chronic skin diseases, such as AD, allergic contact dermatitis (ACD), rosacea, psoriasis, and chronic idiopathic urticaria [84,85,86].

**Figure 5:**
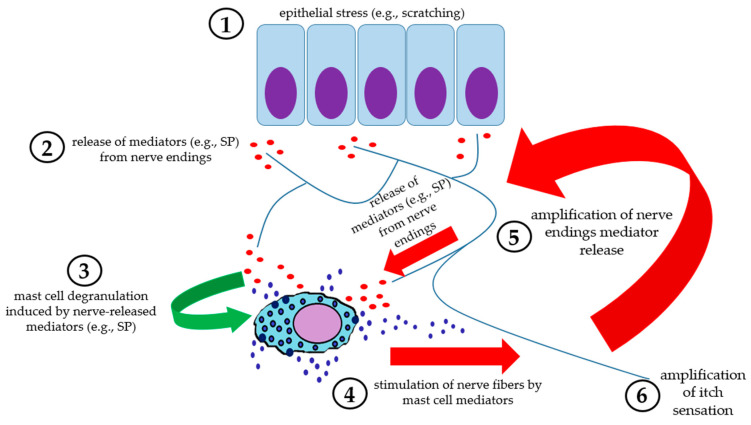
Schematic representation of neurogenic inflammation in the skin. (1) Epithelial stressors (e.g., scratching) lead to mediator release (among them substance P) from nerve endings (2), which stimulate mast cell degranulation (3). Mast cell mediators, in turn, act on peripheral nerve endings (4), enhancing the release of neuromediators (5), thus fueling a positive mast cell-nerve loop, with amplification of itch sensation (6).

Briefly, SP released from terminals of afferent unmyelinated C-fibers and myelinated A-delta fibers binds to MCs and keratinocytes and, together with CGRP and VIP, is a powerful histamine releaser from MCs. Furthermore, MRGPRX2 determines MC degranulation and, together with TRPA1 and PAR-2, induces itching and inflammation through proinflammatory mediators such as histamine, leukotriene B4, and NGF, the latter also having a direct effect on MC degranulation [87,88,89,90,91] (Figure 5).

Moreover, in acute stress and the absence of previous sensitization, SP, NGF, and NK-1 receptors can lead to neurogenic skin inflammation in mice. In addition, the activation of a homolog hypothalamic–pituitary–adrenal (HPA) axis in the skin induced by stress determines the release of proinflammatory hormones, such as corticotropin-releasing factor (CRF), proopiomelanocortin, ACTH, and α-melanocyte-stimulating hormone, neuropeptides, and neurotrophins, with exacerbation of many dermatological diseases [92,93]. In particular, the stimulation of MCs by CRF produces paracrine and autocrine effects, resulting in neuroinflammation [94].

In mouse models of oxazolone-induced contact dermatitis, Boltz et al. explored the neuroinflammatory role of the neuropeptide galanin [95,96]. Additionally, Liu et al. showed through transcriptomic RNA sequencing differentially expressed genes involved in skin neuroinflammation, itch, and pain in ACD model mice and, in particular, the upregulation of MrgprD among Mrgprs in dorsal root ganglia neurons innervating the inflamed skin [97].

The link between MCs and neurogenic inflammation in ACD could be represented by the higher expression of both MCs and pro-adrenomedullin peptide 12 (PAMP12), a ligand of MRGPRX2, in skin lesions of patients with ACD. In particular, MrgprB2MUT mice showed differences in immune cell recruitment, itch behavior, and scratching, suggesting the involvement of IgE/FcεRI-independent pruritogenic pathways [98]. The stimulation of MRGPRX2 by PAMP12 produces the release of tryptase β2 from MCs more than histamine and serotonin. In the skin, tryptase binds to the PAR-2 receptor, with the consequent release of SP and CGRP [99]. In particular, SP elicits inflammatory responses via MRGPRX2 together with cationic peptides released by epithelial cells, eosinophils, and neutrophils [100].

Interestingly, AD is characterized by altered barrier function, additional loss-of-function mutation in filaggrin, and colonization by exotoxin-producing bacteria such as Staphylococcus aureus—where enterotoxins act as superantigens on polyclonal IgE production—while valid experimental models of AD need distinctive “neurodermatitis” features as well as sensory innervation, neurogenic skin inflammation, and AD-like skin lesions triggered by stress [101,102]. In fact, in AD, the number of MCs, expression of SP, PAR2, and genes encoding MRGPRX2 were shown to be increased or upregulated; in particular, S. aureus-related MC degranulation via MRGPRX2 may be induced by δ-toxin or human β-defensin 2 from keratinocytes [71]. Furthermore, TSLP, aberrant in AD, mostly expressed by epithelial cells and in Th2-driven diseases of the skin and lung, has been shown to degranulate MCs through MRGPRX2 in a STAT5-dependent and JNK-supported manner [103].

Also, in rosacea, the aberrant activation and degranulation of cutaneous MCs seem to be linked to MRGPRX2 due to the overexpressed host defense peptide LL-37, leading to the typical flushing, erythema, burning sensation, and itching [104,105]. Overall, in chronic dermatitis, after activation of pruritogenic receptors, exogenous and endogenous pruritogen stimuli are conveyed, in particular through unmyelinated C fibers to the contralateral spinothalamic tract, thalamus, and cortex. CB1 and CB2 are G-protein coupled receptors implicated in neurogenic and inflammatory pain and itch; they are both expressed on peripheral cutaneous nerve fibers and MCs, so they may influence the recruitment of immune cells and skin inflammation in models of dermatitis, and their agonism showed antipruritic effects at central and peripheral levels in systemic and dermatologic diseases, as well as AD and ACD [106].

On the other hand, among transient receptor potential (TRP) ion channels, TRP vanilloid 1 (TRPV1) and TRP ankyrin 1 (TRPA1) appeared to have a role in neurogenic inflammation and itch sensation and can interact with cannabinoids [107].

## 8. Conclusions, Limits, and Perspectives

To summarize, non-immunological and immunological mechanisms, both IgE-mediated and non-IgE-mediated, converge in MCs, providing a modern view of the concepts of pseudoallergy and neurogenic inflammation. Therefore, both in respiratory and cutaneous diseases, with allergic and non-allergic backgrounds, as well as in all organs potentially involved by MC neuroimmune interactions, physiological and pathophysiological mechanisms should be revisited and integrated based on this perspective, with implications for diagnosis, management, and therapy.

The novel acquisitions on MRGPRX2 highlight two particular concepts: the timing and the grade of severity of adverse reactions. From a temporal perspective, preliminary data on the activation and degranulation mechanisms of MCs emphasize the particular rapidity of the MRGPRX2-mediated pathway. Moreover, the clinical manifestations associated with this pathway seem to be more frequently linked to mild-to-moderate reactions. These considerations could therefore suggest a possible clinical phenotype of relatively frequent and particularly early adverse reactions among the so-called immediate adverse reactions, probably not only referred to drugs but also to food allergens, where neurogenic inflammation may contribute to amplifying, maintaining, and transferring the pseudoallergic inflammation.

Neurogenic inflammation, after all, can be modulated, on one hand, by avoiding the formation of endothelial gaps with anti-inflammatory drugs, including steroids, and on the other hand, by preventing the stimulation of cutaneous peripheral sensory nerves, acting on neuropeptide release or by blocking neuropeptide receptors, including inhibitory receptors of MCs. Thus, antagonists of MRGPRX2 to act on MC degranulation and pseudoallergic and neuroinflammatory-related aspects could be useful. Although several potential antagonists have been reported in the literature, including isoflavones and flavonols such as genistein and quercetin, isoliquiritigenin from licorice, piperine from black pepper, and shikonin from Chinese herbal medicine, to our knowledge, there are currently no clinical trials in humans. Moreover, the differences between MRGPRX2 and the rodent ortholog MrgprB2, with only ~53% overall sequence similarity, make setting up models for preclinical studies more complex [108,109].

An MRGPRX2-targeting antagonistic DNA aptamer was studied by Suzuki et al. in rats, with a reduction in histamine release and potential implications in perioperative anaphylaxis [110]. In the context of the development of new molecules [111,112], interestingly, Wollam et al. have very recently identified MRGPRX2 small molecule antagonists inhibiting MC degranulation in vitro, in vivo, and ex vivo in human skin [113].

To date, preclinical trials with GE0117/GE1109/GE1111, EP262/EP9907, and compound B, targeting MRGPRX2, are providing evidence of MC inhibition by MRGPRX2 ligands and block of MRGPRX2-driven MC degranulation in IgE-independent MC activation or MC-driven skin disorders [114,115,116]. In addition, the anti-Kit antibody barzolvolimab and Bruton’s tyrosine kinase inhibitor remibrutinib are being considered in chronic inducible and spontaneous urticaria, respectively, in phase 3 trials [117,118].

Unlike MC stabilizers such as disodium cromoglycate [119], among inhibitory receptors of MCs (Table 1), lirentelimab is an example of a humanized anti-Siglec-8 monoclonal antibody under study [120], while among FDA-approved monoclonal antibodies, omalizumab and dupilumab can interfere with MC degranulation, acting on IgE or IL-4/IL-13 signaling, respectively [18]. In this regard, in addition to the direct effects of known drugs, indirect actions on pathways connected with MCs must also be considered. For example, omalizumab shows the ability to reduce TNF-α and, consequently, the interaction of MCs with granulocytes [121,122], while mepolizumab and benralizumab, targeting IL-5 or its receptor IL-5Rα, respectively, can decrease blood MC progenitors in patients with severe asthma [123].

In chronic dermatitis, the interaction of tryptase β2 with protease-activated receptors on sensory neurons and the effects of SP release may lead to pruritogenic stimulation in a histamine and IgE-independent manner, limiting anti-histamine effects on this neuronal axis. So, cannabinoid-based treatments could help to act on the neurogenic counterpart with a prevalent interest in non-psychoactive non-THC cannabinoids, as well as palmitoylethanolamide (PEA) and cannabidiol (CBD) antagonizing and desensitizing TRPV1 with anti-pruritic effects [124]. Their lipophilic properties can be applied to topical transdermal applications; in fact, clinical trials with PEA or the analog adelmidrol topical treatments have already shown benefits in AD, asteatotic eczema, uremic, and chronic pruritus, [125,126,127], although only observational, open-label, or small patient studies are available [128]. A few cases have utilized randomized or double-blind studies [129,130], so further controlled trials are needed to confirm the efficacy of cannabinoid topical treatments. Moreover, among other formulations, oral administration of cannabinoids is influenced by the first-pass metabolism in the liver and may be exposed to the risk of an overdose [131].

Similarly, TRPV1, TRPV2, NK-1, and CGRP receptors represent other possible targets of neurogenic inflammation, having shown therapeutic potential in experimental pain models [132]. Asivatrep is a potent and selective TRPV1 antagonist that in the phase 3 trial showed, as topical treatment of AD, a significant change in patient-reported pruritus VAS scores compared to vehicle cream [133].

In conclusion, MC is a cell characterized by many surface receptors reflecting its immune and non-immune functions in health and disease in multiple organs and systems. The granule content and timing of release and synthesis determine their role in allergic inflammation, chronic remodeling, and fibrosis processes. MC-mediated clinical allergy correlates may range from rhinitis, bronchial asthma, urticaria-angioedema, and anaphylaxis or may include more extreme situations such as Kounis syndrome, biphasic or idiopathic anaphylaxis.

Alongside the well-known mechanisms of IgE-mediated reactions, the MRGPRX2 receptor expressed on skin mast cells provides an important element in the definition of the mechanisms of mast cell degranulation in response to allergic-like stimuli, in particular pharmacological ones such as contrast agents, neuromuscular blockers, morphine, vancomycin and fluoroquinolones, or in allergic phenotype conditions independent by IgE/FcεRI axis or possibly sustained by mastocytosis, HαT and MCAS. Despite having some similarities with the molecular pathway of FcεRI aggregation, MRGPRX2-induced MC degranulation has been shown to be more rapid, with implications for possible phenotyping in immediate reactions, such as in dose-dependent drug reactions in naïve patients and/or type 2 comorbidities.

Among their heterogeneity, plasticity, and transdifferentiating properties, MCs in the lung can lead to inflammatory or fibrotic diseases as well as nasal polyposis and NARESMA. On the other hand, the close connection with nerve endings and the interaction with takinins via MRGPRX2, primarily SP, make the MC the key cell in the neuroimmune interaction that characterizes the neurogenic inflammation demonstrated in several organs, as well as in lung and skin.

MRGPRX2 antagonists or monoclonal antibodies acting on inhibitory receptors of MCs seem to be potential therapeutic perspectives in modulating both pseudoallergy and neurogenic inflammation, whereas clinical trials with cannabinoids such as PEA and CBD seem to be promising on non-histaminergic pruritus of chronic dermatitis.

Despite the current preliminary experimental data and perspectives, especially in dermatological diseases, it would be interesting to identify biomarkers to discriminate the type of MC degranulation pathway and receptors triggered to guide future pharmacological research.

However, the limitation of this study is that it does not provide a definitive therapeutic conclusion with respect to basic research notions and ongoing clinical trials. The state of the art in pharmacological terms is, after all, the result of the delay that has historically characterized the discovery and progression of mast cell research due to the technical problems previously mentioned. Moreover, the prevalent expression of MRGPRX2 in human skin MCs limits the application of pharmacological research acquisitions to lung diseases. In fact, to our knowledge, except for inhaled anticholinergics and corticosteroids, no sufficient clinical evidence is available to treat and control neurogenic inflammation in asthma.

Therefore, although relevant data have emerged on the characterization of the MRGPRX2-mediated MC degranulation pathway, further studies are needed to better understand when and how IgE or MRGPRX2-mediated mechanisms are activated in vivo, how they intersect with each other, and to identify new therapeutic strategies for MC-mediated neuroimmune inflammation, particularly in the lung.

## Figures and Tables

**Table 1:** Main mast cell surface receptors and neuroimmune interactions.

Receptor	Main Ligand	Function
KIT (CD117)	SCF	Proliferation, maturation, and survival of MCs *D816V mutation or other activating KIT mutations in mastocytosis*
TLRs, NLRs RLRs	PAMPs	Recognition of Pathogen Associated Molecular Patterns. May induce MC degranulation
FcεRI	IgE high-affinity receptor	Allergy, parasitic infection, and regulation of IgE levels
FcεRII	IgE low-affinity receptor	
MRGPRX2	SP, CGRP, cationic peptides released from eosinophils, neutrophils, and epithelial cells, and cationic drugs	Pseudoallergy Neurogenic Inflammation
CB1 CB2	Exogenous and endogenous cannabinoids	Cannabinoid System Inflammatory pain and itch
TRPA1	Bradykinin and cannabinoids	
TRPV1	Capsaicin and cannabinoids	
TRPV2	Cannabinoids Mechanical, thermal, Osmotic, and laser light stimulation	
ADGRE 2 (EMR2) *C492Y mutation*	ADGRE2 ligand dermatan sulfate	Response to physical stimuli Physical urticaria *C492Y mutant ADGRE2 in autosominal dominant vibratory urticaria and HαT*
NOX2	UVA irradiation	
ST2 receptors	IL-33	Receptors for alarmins *Implications in MCAS*
P2X1	ATP	
Siglec-8 Siglec-6	Sialic acid-binding immunoglobulin-type lectins	Inhibitory receptors Inhibition of MC degranulation
CD200R	CD 200	
CD300a	Phosphatidylserine/phosphatidylethanolamine	
FcRIIb	Low-affinity inhibitory receptor for the Fc region of IgG.	

## Data Availability

Not applicable.
